# Associations Between Isokinetic Torque and External Load Metrics During Youth Soccer Matches

**DOI:** 10.3390/sports13090311

**Published:** 2025-09-08

**Authors:** Yiannis Michailidis

**Affiliations:** Laboratory of Evaluation of Human Biological Performance, Department of Physical Education and Sports Sciences, Aristotle University of Thessaloniki, 57001 Thessaloniki, Greece; ioannimd@phed.auth.gr; Tel.: +30-231-099-2248

**Keywords:** correlation, strength, football, GPS, running performance, association

## Abstract

Physical fitness, especially strength, is key for football performance and injury prevention, but its role in match running remains unclear. This study examined correlations between knee flexor and extensor isokinetic torque (60°/s, 180°/s, 300°/s) and running performance in 20 U17 players (age 16 ± 0.5 years, height 1.78 ± 0.05 m, weight 71 ± 7.1 kg) across 16 matches, with GPS tracking (Apex, STATSports, Newry, Northern Ireland). Results varied by position. Central defenders showed a negative correlation between non-dominant knee flexor torque at 300°/s and high-speed running distance (r = −0.975, *p* = 0.025). Side defenders displayed positive correlations between dominant knee flexor torque at 300°/s and both total distance and moderate running (r = 0.885–0.976, *p* < 0.05), but negative correlations with maximum speed (r < −0.89, *p* < 0.05). Central midfielders had several negative associations between dominant knee flexor torque at 300°/s and accelerations or decelerations (r < −0.88, *p* < 0.05). Side midfielders and forwards showed positive correlations between torque at higher speeds (180°/s, 300°/s) and sprint distance (r ≥ 0.85, *p* < 0.05). Overall, associations differed by position, velocity, and performance variable, reflecting tactical demands and the limits of single-joint testing. However, in SMFs, high positive correlations were observed, highlighting the importance of strength and its necessity in the training process.

## 1. Introduction

Football is a highly popular sport involving millions of athletes of various levels and ages [1,2]. Player performance depends on various factors such as technique, tactics, physical condition, etc., [3], which are developed over a long-term training process.

In modern football, physical fitness is particularly important for players to cope with the increasing pace of matches, as the frequency of high- and maximum-intensity actions—as well as player duels—has increased [4]. Physical fitness in football is not one-dimensional; a modern footballer must have highly developed aerobic capacity (endurance) as well as anaerobic capacity (strength, speed, power) [5].

One of the physical abilities of particular importance for football performance is strength, specifically the form known as power (explosive strength) [6,7]. Most high-intensity actions during a match—such as jumps, accelerations, decelerations, sprints, and duels—require the application of high levels of force in a short amount of time (high power). Power is a key attribute for all modern footballers, as it is directly related to the effectiveness of both their defensive and offensive actions [8].

Strength also influences a player’s running economy. Running economy is defined as the oxygen or energy required to cover a certain distance or to maintain a specific submaximal running speed [9]. Recent studies have observed that strength training can improve running economy [10,11,12,13], particularly influencing running time trial performance [14,15]. Therefore, a similar improvement in footballers could enhance their running performance during matches, as they cover approximately 10–12 km per game, 2–3 km of which are at high speeds.

In recent decades, technological advances (global positioning systems) have enabled monitoring of the external load of footballers during matches [16,17]. From the GPS variables, the analysis included the distances covered by the players in zone 14.4–19.79 km/h, zone 19.8–25.19 km/h, zone ≥ 25.2 km/h), and the total distance covered. Also included were accelerations with an intensity between 2 and 3 m/s^2^, above 3 m/s^2^, as well as decelerations with an intensity between −3 and −2 m/s^2^ and below −3 m/s^2^. In addition, the player’s maximum speed and match pace were used.

As mentioned above, several variables measured using GPS may be influenced by a player’s strength level (e.g., maximum speed, accelerations, decelerations, distance covered at high intensity, etc.). Biomechanically, greater isokinetic strength in knee flexors and extensors enhances the ability to generate rapid, forceful contractions necessary for accelerations, decelerations, and changes in direction [18]. Stronger muscles improve joint stability and reduce injury risk, allowing players to maintain performance during demanding actions [19]. Physiologically, increased muscle strength contributes to improved neuromuscular efficiency and power output, which supports better sprinting speed and endurance by delaying fatigue onset during repeated high-intensity efforts [20,21]. Moreover, enhanced strength facilitates more effective energy transfer and reduces metabolic cost during running, thereby positively influencing total distance covered and high-speed running variables [22]. However, a review of the literature reveals a significant research gap, as only few studies [23,24,25] have investigated the relationship between strength parameters and running performance. Most studies focus on examining the relationship between strength and other physical abilities assessed via laboratory or field tests [26,27].

The difference in methodology between these studies is particularly important: in one case, correlations are sought between specific physical abilities (e.g., power and speed), which are easier to observe—especially when based on the same physiological mechanism (e.g., stretch-shortening cycle—jump ability and sprinting); in the other case, correlations are sought with indicators of match running performance, i.e., metrics used to evaluate football performance. Observing such relationships could enhance training effectiveness, as strength elements with a direct impact on in-game running performance would be specifically targeted.

Therefore, the aim of the present study was to investigate the relationship between the isokinetic torque of the knee flexors and extensors of young footballers and external load indicators during official matches. Based on the study objectives, we hypothesize that isokinetic torque of the knee flexor and extensor muscles will be positively correlated with key match running performance metrics, including maximum speed, high-speed running distance, and the number of accelerations and decelerations. Furthermore, we expect these correlations to vary according to playing position and angular velocity of assessment, with stronger associations anticipated at higher testing velocities and in positions that demand greater sprinting and change-of-direction activities, such as side defenders, side midfielders and forwards.

## 2. Materials and Methods

### 2.1. Participants

To conduct the study, 26 players from the under-17 team of a football academy were invited. Of these, only 20 footballers participated in the study (age 16.0 ± 0.5 years, training age 11.1 ± 1.3 years, height 180 ± 10 cm, weight 71.0 ± 7.1 kg, body fat percentage 11.6 ± 1.3), all of whom met the inclusion criteria, which were: (a) absence of injuries in the past month, (b) no use of medication, (c) participation in all pre-match tests (anthropometrics, isokinetic torque at three speeds: 60°/s, 180°/s, 300°/s), and (d) participation in at least 85 min of the match. The team competed in the national championship of its country and participated in 4 training sessions and 1 official match per week. The players were categorized based on their playing position as central defenders (CD, n = 4, 20%), side defenders (SD, n = 5, 25%), central midfielders (CM, n = 5, 25%), side midfielders-forwards (SMF, n = 6, 30%). Informed consent was obtained from all subjects and their parents involved in the study. The local institutional review board approved the study (approval number 217/2024) in accordance with the Helsinki Declaration.

### 2.2. Procedure

The footballers were already familiar with the tests, as these were part of the routine assessments conducted during their previous years of ergometric evaluations. The study was conducted over the course of the first 16 matches of the competitive season. All tests were conducted in the morning, between 9:00 and 12:00 AM, to control circadian variations in performance, within a laboratory setting maintaining stable temperature (~22 °C) and humidity (~40%) conditions. At the end of the preseason, the players visited the laboratory, where anthropometric measurements were taken first. This was followed by a 10 min warm-up (7 min of cycling and 3 min of stretching), after which the isokinetic torque assessment was carried out. During the procedure, participants were allowed to drink water ad libitumto ensure proper hydration during testing. Subsequently, once the competitive season began, the players’ external load during the first 16 matches was recorded using GPS technology. Dominant and non-dominant legs were determined based on player self-report, with the dominant leg defined as the preferred limb for ball control and manipulation during match play.

### 2.3. Anthropometric Measurements

The players’ body height was measured using a stadiometer with 0.1 cm precision (Seca 220e, Hamburg, Germany). Body weight and body fat percentage were measured using a dual-frequency body composition analyzer (TANITA DC-360, Tanita Corporation, Tokyo, Japan), based on the principles of bioelectrical impedance.

### 2.4. Isokinetic Strength Testing

Muscular strength of the hamstrings and quadriceps were evaluated under isokinetic conditions using a Cybex II dynamometer (Lumex Inc., Ronkonkoma, NY, USA). At each test velocity, the highest torque output was recorded, with corrections applied to exclude the effects of limb mass and equipment resistance. Participants were seated in a modifiable chair attached to the device, with stabilizing straps placed across the torso, hips, and thighs to restrict extraneous movements. The test leg was set at 90 degrees of knee flexion (where 0 degrees denotes full extension), ensuring proper alignment between the knee joint axis and the lever arm’s axis at the distal lateral femoral condyle. Lever arm length and resistance pad position were adjusted individually, with the pad placed just above the medial malleolus. The opposite leg remained passive, unsupported.

Movements were executed in a fixed pattern: extension began from 90° flexion, while flexion started from full extension. Participants were instructed to exert maximal effort, moving their leg through the complete range of motion as quickly and forcefully as possible while maintaining crossed arms. Three trials were performed at each test speed, and the peak torque value was captured for analysis. Recovery between repetitions was set at 30 s, with one-minute intervals between velocity changes. Verbal cues were provided throughout to encourage full voluntary effort. Measurements were taken at speeds of 60°, 180°, and 300° per second which are reported to represent different strength qualities [28,29]. Specifically, testing at 60°/s reflects maximal strength, at 180°/s reflects muscular endurance, and at 300°/s reflects power. The purpose of selecting these three different velocities was to provide a comprehensive profile of strength capacity in young footballers and to examine the relevance of each strength quality to match-running performance variables. Peak isokinetic torque was considered the highest force output during the complete motion range for both muscle groups [28]. To assess muscle balance, the conventional hamstring-to-quadriceps (H:Q) ratio was determined by dividing the maximal concentric torque of the hamstrings (flexion) by that of the quadriceps (extension) at each velocity. All measurements were performed by the same person.

### 2.5. Global Positioning System Variables

To record the external load during the matches, the global navigation satellite system 10 Hz Apex (STATSports, Newry, Northern Ireland) was used. Previous studies have reported that the above GPS system is valid and reliable for measuring the external load of football players [30,31]. The device was placed in a special vest between the player’s shoulder blades and was activated at the beginning of each half and deactivated at the end of each half. After the matches, the transmitters were placed in a special company docking station to transfer the data from the devices to the computer, from which the data were exported in CSV format. Each player always used the same GPS unit. The distances covered by players were categorized into three speed zones to capture different intensities of running performance during the match.

Zone 1 (14.4–19.79 km/h): This zone represents moderate-intensity running and is often associated with sustained efforts during the game, such as jogging or controlled running. Distances covered in this zone provide insights into the player’s ability to maintain movement over prolonged periods without reaching maximal effort, reflecting aerobic endurance components.

Zone 2 (19.8–25.19 km/h): This zone corresponds to high-speed running (HSR), encompassing efforts that require substantial anaerobic capacity and neuromuscular power. Running at these speeds typically involves crucial phases such as offensive and defensive transitions, where players need to accelerate rapidly and cover ground effectively [32].

Zone 3 (≥25.2 km/h): This zone captures sprinting efforts, representing maximal or near-maximal running speeds. Sprint distance is critical in football for decisive actions like breakaways, counters, and defensive recoveries. Due to the intermittent and high-intensity nature of these efforts, sprinting distance is highly sensitive to fatigue and training status, making it an important variable for assessing player readiness and physical condition [33].

Total Distance (TD): The total distance covered during the match sums all running intensities and provides a comprehensive measure of overall workload. TD is a fundamental variable frequently used to gauge player activity levels and has a low coefficient of variation between matches, enhancing its utility for monitoring and comparison purposes [34].

Accelerations between 2 and 3 m/s^2^ and above 3 m/s^2^: Accelerations reflect the ability of a player to rapidly increase running speed. The inclusion of two intensity categories allows differentiation between moderate- and high-intensity accelerative efforts. These actions are mechanically demanding and involve explosive muscle contractions, contributing significantly to match performance. Previous research indicates that acceleration counts correlate well with neuromuscular strength and power measures, supporting their relevance in performance analysis [33].

Decelerations between −3 and −2 m/s^2^ and below −3 m/s^2^: Decelerations represent the capacity to reduce speed rapidly, a critical component for change-of-direction and tactical positioning. Like accelerations, decelerations were categorized into moderate and high intensities to better characterize the physical demands placed on players. Previous research indicates correlations between strength and deceleration ability [35].

Maximum running speed attained during the match serves as an indicator of a player’s top-end velocity capacity. It is a critical determinant of match-defining moments such as successful sprints and defensive recoveries. Maximum speed measurements are reliable and have been shown to correlate with strength and power metrics, making this variable valuable for evaluating player explosive capabilities [36].

Match pace was evaluated through variables such as total distance per minute (TD/min) and high-speed running distance above 19.8 km/h. These metrics provide insights into the intensity of activity relative to playing time and overall workload efficiency. Match pace is useful for standardizing comparisons between players with different playing durations.

### 2.6. Statistical Analysis

To assess the normal distribution of the data, the Shapiro–Wilk test was conducted, revealing that parametric statistical tests could be applied. Pearson correlation test was applied to examine potential correlations between the isokinetic strength indexes with GPS running performance indexes during matches. The strength of the correlation was determined based on the value of r: r ≤ 0.1, trivial; 0.1 < r ≤ 0.3, small; 0.3 < r ≤ 0.5, medium; 0.5 < r ≤ 0.7, large; 0.7 < r ≤ 0.9, very large; and r > 0.9, almost perfect. Correlation heatmaps were performed using JASP version 0.19.3.0 for Windows (ASP Team: Amsterdam, The Netherlands) [37]. Statistical significance was set at *p* < 0.05. All analyses were performed using SPSS version 28.0 (SPSS, Inc., Chicago, IL, USA).

## 3. Results

**Figure 1:**
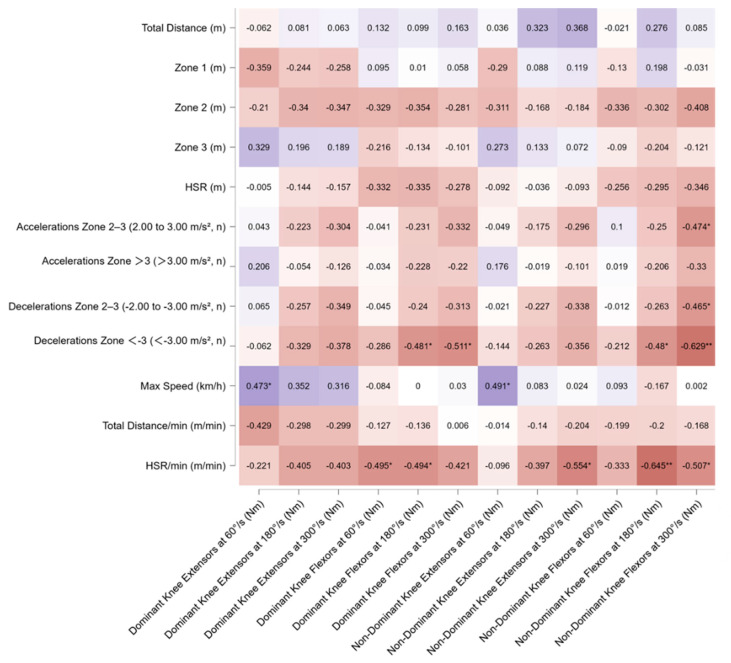
Pearson’s correlation matrix of isokinetic variables of dominant and non-dominant extensors and flexors leg muscles across different angular velocities (60°/s, 180°/s, 300°/s) with match running variables for all players. The heatmap displays Pearson’s correlation coefficients (*r*) between isokinetic variables of dominant and non-dominant extensors and flexors leg muscles across different angular velocities (60°/s, 180°/s, 300°/s) with match running variables for all players. Significant correlations are indicated with * *p* < 0.05, ** *p* < 0.01,. Warmer colors indicate negative correlations, while cooler colors represent positive correlations. Note: Significant relationships were observed between knee flexor strength and deceleration metrics, particularly for decelerations greater than −3.00 m/s^2^ (r = −0.48 to −0.63, *p* < 0.01). Dominant knee extensor strength at 60°/s correlated positively with maximum speed (r = 0.47, *p* < 0.05). Additionally, negative correlations were identified between knee flexor strength at higher angular velocities and relative high-speed running per minute (r = −0.49 to −0.65, *p* < 0.01).

From the correlation analysis of all players combined, negative associations were observed for the knee flexors of the dominant limb at 60° and 180° (r = −0.495, *p* < 0.05 and r = −0.494, *p* < 0.05, respectively) and for the non-dominant limb at 180° and 300° with HSR/min (r = −0.645, *p* < 0.01 and r = −0.507, *p* < 0.05, respectively). Negative correlations were also found between the flexors of the dominant and non-dominant limb at 180° and 300° with the number of high-intensity decelerations (r = −0.481, *p* < 0.05 and r = −0.511, *p* < 0.05; r = −0.480, *p* < 0.05 and r = −0.629, *p* < 0.01, respectively). All correlations are presented in Figure 1.

**Figure 2:**
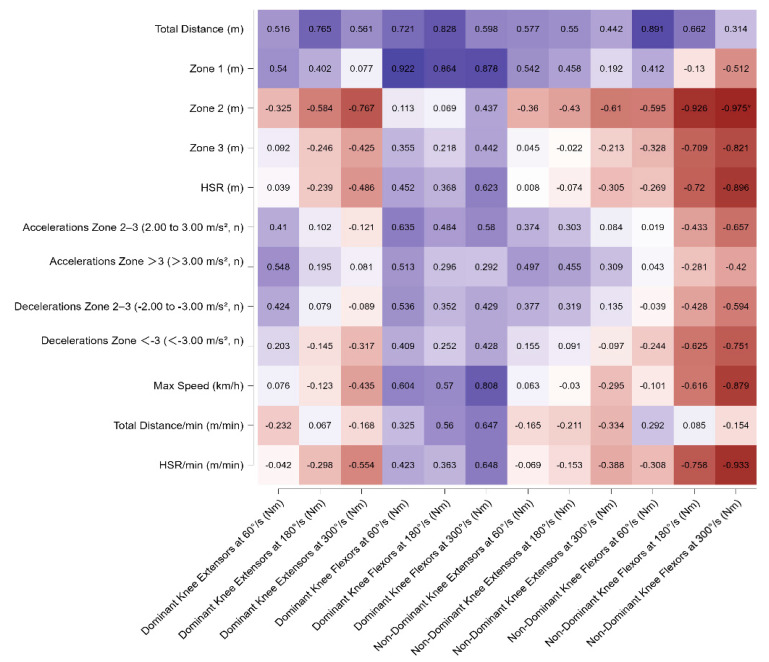
Pearson’s correlation matrix of isokinetic variables of dominant and non-dominant extensors and flexors leg muscles across different angular velocities (60°/s, 180°/s, 300°/s) with match running variables for central defenders. The heatmap displays Pearson’s correlation coefficients (*r*) between isokinetic variables of dominant and non-dominant extensors and flexors leg muscles across different angular velocities (60°/s, 180°/s, 300°/s) with match running variables for central defenders. Significant correlations are indicated with * *p* < 0.05. Warmer colors indicate negative correlations, while cooler colors represent positive correlations. Note: For CD, significant relationships were observed between GPS-derived external load indicators and isokinetic strength parameters. Strong positive correlations were found between total distance, Zone 1, HSR, and accelerations with dominant and non-dominant knee extension peak torque at 180°/s and 300°/s (r = 0.51–0.92, *p* < 0.01). Max speed also correlated strongly with dominant and non-dominant knee extensors at 180°/s (r = 0.57–0.81, *p* < 0.01). Conversely, Zone 2 running and HSR demonstrated strong negative correlations with knee flexor strength, particularly in non-dominant assessments at 180°/s and 300°/s (r = −0.59 to −0.98, *p* < 0.01).

In central defenders (CD), a strong negative correlation was observed between the distance covered in zone 2 and the knee flexors of the non-dominant limb at 300°/s. No other significant associations were detected. All results of the statistical analysis are presented in Figure 2.

**Figure 3:**
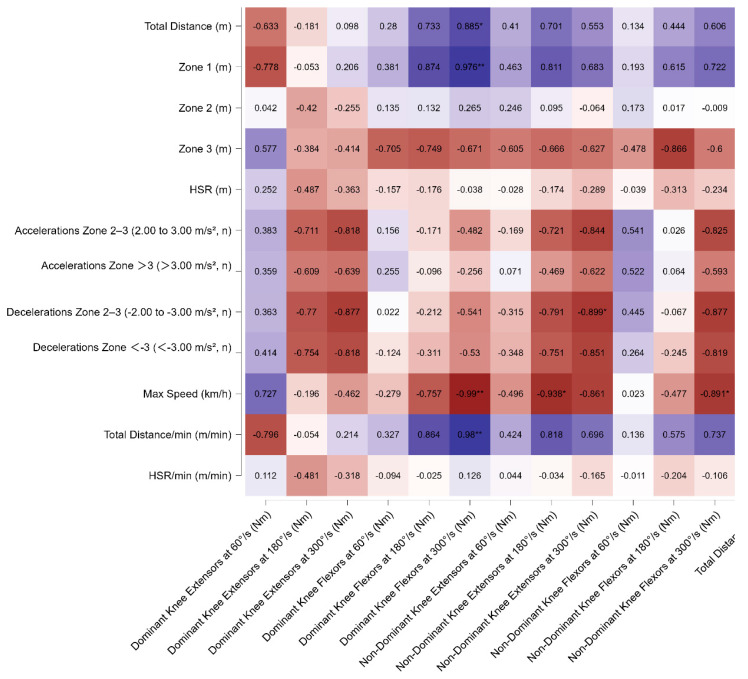
Pearson’s correlation matrix of isokinetic variables of dominant and non-dominant extensors and flexors leg muscles across different angular velocities (60°/s, 180°/s, 300°/s) with match running variables for side defenders. The heatmap displays Pearson’s correlation coefficients (*r*) between isokinetic variables of dominant and non-dominant extensors and flexors leg muscles across different angular velocities (60°/s, 180°/s, 300°/s) with match running variables for side defenders. Significant correlations are indicated with * *p* < 0.05, ** *p* < 0.01. Warmer colors indicate negative correlations, while cooler colors represent positive correlations. Note: For SD—Significant relationships were observed between GPS-derived external load indicators and isokinetic strength parameters. Strong positive correlations were found between total distance, total distance per minute, maximal speed, and both dominant and non-dominant knee extension torque at 180°/s and 300°/s (r = 0.73–0.99, *p* < 0.01). Zone 1 activity also correlated very strongly with knee extensor strength (r = 0.87–0.98, *p* < 0.01). In contrast, strong negative correlations were observed between accelerations (2–3 m/s^2^ and >3 m/s^2^) and decelerations (−2 to −3 m/s^2^ and <−3 m/s^2^) with dominant and non-dominant knee flexor strength at higher angular velocities (r = −0.59 to −0.90, *p* < 0.01).

In side defenders (SD), strong positive correlations were observed between the flexors of the dominant limb at 300°/s and both TD and distance covered in zone 1 (representing tactical positioning running) (r = 0.885, *p* = 0.046 and r = 0.976, *p* = 0.004, respectively). Moreover, a strong negative correlation was observed between the flexors of both limbs at 300°/s and maximum speed (r < −0.89, *p* < 0.05). A summary of these findings is presented in Figure 3.

**Figure 4:**
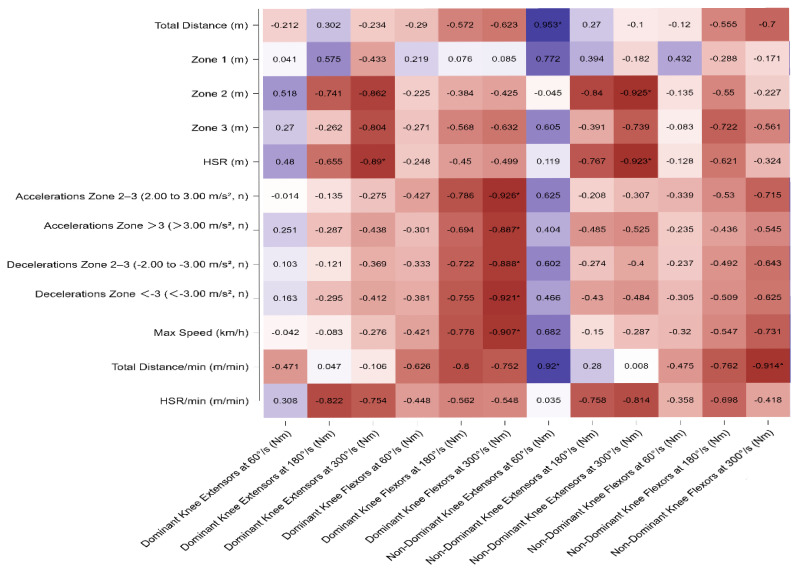
Pearson’s correlation matrix of isokinetic variables of dominant and non-dominant extensors and flexors leg muscles across different angular velocities (60°/s, 180°/s, 300°/s) with match running variables for central midfielders. The heatmap displays Pearson’s correlation coefficients (*r*) between isokinetic variables of dominant and non-dominant extensors and flexors leg muscles across different angular velocities (60°/s, 180°/s, 300°/s) with match running variables for central midfielders. Significant correlations are indicated with * *p* < 0.05. Warmer colors indicate negative correlations, while cooler colors represent positive correlations. Note: For CM—Significant relationships were observed between GPS-derived external load indicators and isokinetic strength parameters. Total distance and total distance/min showed very strong positive correlations with dominant knee extensors at 60°/s (r = 0.953 and r = 0.920, *p* < 0.01). HSR/min demonstrated strong negative associations with dominant knee extensors at 180°/s (r = −0.822, *p* < 0.01) and non-dominant knee extensors at 180°/s (r = −0.758, *p* < 0.01). Additionally, accelerations >3 m/s^2^ and decelerations <−3 m/s^2^ were strongly negatively correlated with dominant knee flexors at 300°/s (r = −0.887 and r = −0.881, *p* < 0.01).

In central midfielders (CM), strong negative correlations were found between the extensors of both the dominant and non-dominant limbs at 300°/s and HSR (r = −0.890, *p* = 0.043 and r = −0.923, *p* = 0.025, respectively). Furthermore, the knee flexors of the dominant limb at 300°/s were negatively correlated with all categories of accelerations and decelerations as well as with maximum speed (r < −0.88, *p* < 0.05). A summary of these results is presented in Figure 4.

**Figure 5:**
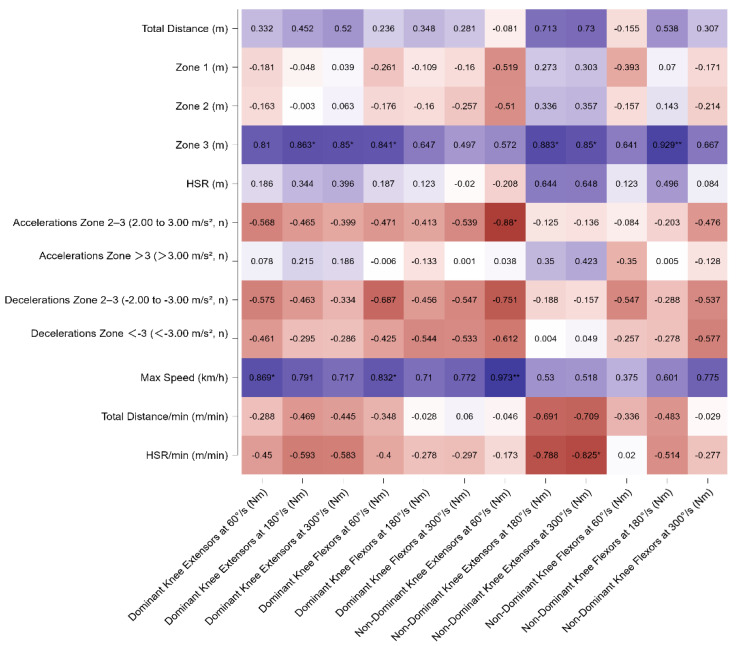
Pearson’s correlation matrix of isokinetic variables of dominant and non-dominant extensors and flexors leg muscles across different angular velocities (60°/s, 180°/s, 300°/s) with match running variables for side midfielders and forwards. The heatmap displays Pearson’s correlation coefficients (*r*) between isokinetic variables of dominant and non-dominant extensors and flexors leg muscles across different angular velocities (60°/s, 180°/s, 300°/s) with match running variables for side midfielders and forwards. Significant correlations are indicated with * *p* < 0.05, ** *p* < 0.01. Warmer colors indicate negative correlations, while cooler colors represent positive correlations. Note: For F and SM—Significant relationships were observed between GPS-derived external load indicators and isokinetic strength parameters. Strong positive correlations were found between Zone 3 activity and both dominant and non-dominant knee extension torque at 180°/s and 300°/s (r = 0.81–0.93, *p* < 0.01). Maximal speed correlated strongly with knee extensors at 60°/s, 180°/s, and 300°/s in both limbs (r = 0.77–0.97, *p* < 0.01). Additionally, negative correlations were observed between accelerations (2–3 m/s^2^) and decelerations (−2 to −3 m/s^2^; <−3 m/s^2^) with knee flexor strength, particularly in non-dominant assessments at higher angular velocities (r = −0.47 to −0.82, *p* < 0.01). Table 1 presents a consolidated overview of all strong correlations identified in this study.

In side midfielders (SMF), positive correlations were observed between the extensors of both limbs at 180° and 300°/s and sprint distance (r > 0.84, *p* < 0.05). In addition, strong positive correlations were found between the flexors and extensors of the dominant limb at 60°/s and maximum speed (r = 0.869, *p* = 0.025 and r = 0.832, *p* = 0.040, respectively) (Figure 5).

**Table 1:** Major correlations between lower-limb isokinetic torque at different angular velocities and running load parameters.

Position	GPS Variable	Strength Variable	r	*p*	95% CI		ES	SE ES
					Lower	Upper		
CD	Zone 2 (m)	Non-Dominant Knee Flexors at 300°/s (Nm)	−0.975	0.025 *	−1.000	−0.228	−2.192	1.000
CM	Total Distance (m)	Non-Dominant Knee Extensors at 300°/s (Nm)	0.953	0.012 *	0.442	0.997	1.861	0.707
CM	Zone 2 (m)	Non-Dominant Knee Flexors at 60°/s (Nm)	−0.925	0.024 *	−0.995	−0.231	−1.621	0.707
CM	HSR (m)	Dominant Knee Extensors at 300°/s (Nm)	−0.890	0.043 *	−0.993	−0.038	−1.424	0.707
CM	HSR (m)	Non-Dominant Knee Extensors at 300°/s (Nm)	−0.923	0.025 *	−0.995	−0.222	−1.612	0.707
CM	Accelerations Zone 2–3 (n)	Dominant Knee Flexors at 300°/s (Nm)	−0.926	0.024 *	−0.995	−0.239	−1.629	0.707
CM	Accelerations Zone >3 (n)	Dominant Knee Flexors at 300°/s (Nm)	−0.887	0.045 *	−0.993	−0.023	−1.409	0.707
CM	Decelerations Zone 2–3 (n)	Dominant Knee Flexors at 300°/s (Nm)	−0.888	0.044 *	−0.993	−0.028	−1.414	0.707
CM	Decelerations Zone >3 (n)	Dominant Knee Flexors at 300°/s (Nm)	−0.921	0.026 *	−0.995	−0.206	−1.595	0.707
CM	Max Speed (km/h)	Dominant Knee Flexors at 300°/s (Nm)	−0.907	0.034 *	−0.994	−0.124	−1.510	0.707
CM	Total Distance/min (m/min)	Non-Dominant Knee Extensors at 60°/s (Nm)	0.920	0.027 *	0.200	0.995	1.588	0.707
CM	Total Distance/min (m/min)	Non-Dominant Knee Flexors at 300°/s (Nm)	−0.914	0.030 *	−0.994	−0.166	−1.553	0.707
SMF	Zone 3 (m)	Dominant Knee Extensors at 180°/s (Nm)	0.863	0.027 *	0.170	0.985	1.304	0.577
SMF	Zone 3 (m)	Dominant Knee Extensors at 300°/s (Nm)	0.850	0.032 *	0.123	0.983	1.256	0.577
SMF	Zone 3 (m)	Dominant Knee Flexors at 60°/s (Nm)	0.841	0.036 *	0.091	0.982	1.223	0.577
SMF	Zone 3 (m)	Non-Dominant Knee Extensors at 180°/s (Nm)	0.883	0.020 *	0.251	0.987	1.388	0.577
SMF	Zone 3 (m)	Non-Dominant Knee Extensors at 300°/s (Nm)	0.850	0.032 *	0.126	0.983	1.258	0.577
SMF	Zone 3 (m)	Non-Dominant Knee Flexors at 180°/s (Nm)	0.929	0.007 **	0.476	0.992	1.650	0.577
SMF	Accelerations Zone 2–3 (n)	Non-Dominant Knee Extensors at 60°/s (Nm)	−0.880	0.021 *	−0.987	−0.239	−1.375	0.577
SMF	Max Speed (km/h)	Dominant Knee Extensors at 60°/s (Nm)	0.869	0.025 *	0.195	0.986	1.329	0.577
SMF	Max Speed (km/h)	Dominant Knee Flexors at 60°/s (Nm)	0.832	0.040 *	0.062	0.981	1.193	0.577
SMF	Max Speed (km/h)	Non-Dominant Knee Extensors at 60°/s (Nm)	0.973	0.001 ***	0.769	0.997	2.149	0.577
SMF	HSR/min (m/min)	Non-Dominant Knee Extensors at 300°/s (Nm)	−0.825	0.043 *	−0.980	−0.041	−1.173	0.577
SD	Total Distance (m)	Dominant Knee Flexors at 300°/s (Nm)	0.885	0.046 *	0.013	0.992	1.399	0.707
SD	Zone 1 (m)	Dominant Knee Flexors at 300°/s (Nm)	0.976	0.004 **	0.676	0.998	2.208	0.707
SD	Decelerations Zone 2–3 (n)	Non-Dominant Knee Extensors at 300°/s (Nm)	−0.899	0.038 *	−0.993	−0.082	−1.468	0.707
SD	Max Speed (km/h)	Dominant Knee Flexors at 300°/s (Nm)	−0.990	0.001 ***	−0.999	−0.847	−2.632	0.707
SD	Max Speed (km/h)	Non-Dominant Knee Extensors at 180°/s (Nm)	−0.938	0.018 *	−0.996	−0.325	−1.723	0.707
SD	Max Speed (km/h)	Non-Dominant Knee Flexors at 300°/s (Nm)	−0.891	0.042 *	−0.993	−0.041	−1.427	0.707
SD	Total Distance/min (m/min)	Dominant Knee Flexors at 300°/s (Nm)	0.980	0.003 **	0.719	0.999	2.292	0.707

CD, central defenders; SD, side defenders; CM, central midfielders; SMF, side midfielders and forwards; Zone 1 (14.4–19.79 km/h); Zone 2 (19.8–25.19 km/h); Zone 3 (≥25.2 km/h); HSR, high-speed running (>19.8 km/h); ES, effect size; SE ES, standard error effect size; Significant correlations are indicated with * *p* < 0.05, ** *p* < 0.01, *** *p* < 0.001.

## 4. Discussion

The aim of the present study was to investigate the existence of correlations between the isokinetic torque of the lower limbs in young football players and running performance indicators from official matches. The initial hypothesis of the study was partially confirmed, as correlations were found between isokinetic torque and variables such as sprinting, maximum speed, and high-speed running (HSR). However, these correlations did not follow a consistent pattern and appeared to vary depending on playing position, the angular velocity used for isokinetic evaluation, and the limb being tested.

Before discussing the results by playing position, it can be noted that there was no substantial effect of the dominant versus non-dominant limb, as the stronger correlations were evenly distributed between them. The type of correlations (positive vs. negative) was also similarly balanced. However, the angular velocity at which the majority of correlations were observed was 300°/s (19 out of 30). Finally, in SMF players predominantly positive correlations were found, whereas in CM players, negative correlations were more prevalent. This observation may be directly related to the functional role of each playing position, as will be discussed below.

Starting with central defenders (CDs), it was observed that higher isokinetic torque at 300°/s (power) was negatively correlated with high-speed running distance. This finding contradicts the initial hypothesis and may be better understood by considering the tactical role of this playing position. Previous research has shown that CDs cover the least distance in sprints and HSR [38], likely due to the increased defensive responsibilities and high player density in their zones. As previously noted, the literature on this topic is very limited. The most relevant study is that of Pedersen [23], conducted on female footballers, which found no significant correlation between strength (1RM) and match running performance indicators (max speed, accelerations, decelerations) in a friendly match. In that study, players were divided into central and wide roles. Additionally, studies that used jump tests to explore such correlations found no significant relationships [23,25,39]. In another study [40] investigating strength and speed demands in 4v4 and 8v8 small-sided games, a moderate correlation between power and accelerations was found. This is essentially the only study reporting such correlations; however, it should be noted that the smaller the game format (e.g., 4v4), the more frequent the duels and the higher the frequency of decelerations and accelerations—factors which could influence the results.

In side defenders (SDs), the observed correlations were limited to high angular velocities (180–300°/s), with positive correlations associated with TD, pace (TD/min) and distance in zone 1, while negative correlations were associated with high-intensity metrics (decelerations, max speed). Considering the fact that players in this position typically cover large distances, this observation might be explained by the impact of strength training on running economy [10,11,12,13]. It is possible that players with higher isokinetic strength perform better in medium-intensity running activities due to improved running economy. The negative correlations observed between isokinetic torque and high-intensity performance metrics (decelerations, and maximal speed) among side defenders may be explained by the influence of body composition and movement demands on match performance. Higher isokinetic torque values often reflect greater absolute muscle mass, which can increase total body mass and, in turn, reduce relative strength-to-weight ratios. For side defenders—whose tactical role requires frequent high-velocity sprints along the flanks, rapid changes in direction, and repeated accelerations over varying distances—excessive muscle mass may hinder movement efficiency and agility, thereby compromising their ability to perform these explosive actions effectively [41]. Moreover, the high-intensity running demands of this position are often more dependent on neuromuscular coordination, elastic strength, and reactive power than on maximal torque measured in isolated, controlled conditions [28]. As a result, players with greater absolute torque but lower relative power output may demonstrate reduced performance in GPS-derived high-intensity metrics.

For central midfielders (CMs), the positive correlations observed between isokinetic torque at 60°/s and pace TD/min) likely reflect the importance of maximal muscle strength at slower contraction speeds for sustaining prolonged, low-to-moderate-intensity efforts throughout a match. It has also been reported that strength training can improve running economy in well-trained runners, thereby affecting their performance [42]. Conversely, the negative correlations with high-speed running and torque measured at 300°/s suggest that maximal strength at very high contraction velocities may not directly translate to improved performance in explosive sprinting or high-velocity actions. High-speed running demands rapid force production and neuromuscular coordination that are not fully captured by isokinetic torque (single joint) at very high velocities. Furthermore, greater maximal strength at slow speeds may be linked with increased muscle mass, which could potentially impair acceleration and sprinting ability due to increased inertia [43]. This divergence underscores the complex relationship between strength characteristics at different contraction velocities and specific match-running demands. Also, it is important to consider the tactical role of this position. Specifically, these players are involved in all four phases of the game (defense, attack, offensive and defensive transitions), often covering long distances, typically at moderate intensities [16]. Additionally, they operate in areas with high player density and limited space, making it difficult to execute sprints. Their high-intensity efforts are mostly characterized by duels rather than frequent maximal sprints.

Finally, for side midfielders and forwards (SMFs), the study found exclusively positive correlations across all isokinetic testing velocities with high-intensity running performance variables and maximum speed achieved. This finding aligns with the positional demands placed on SMFs, who typically operate in wider and more advanced zones of the pitch where there is greater spatial freedom to execute frequent and intense sprints [44]. Unlike central midfielders (CMs), whose roles often require more frequent changes in direction and shorter bursts within congested areas, SMFs have more opportunities to utilize their maximal speed during offensive transitions and counterattacks [45]. Additionally, wide midfielders are tasked with rapid defensive recovery runs during transitions to disrupt opposition attacks, further emphasizing the need for high-speed and powerful running capacity [46]. The positive correlations suggest that players with higher isokinetic strength—reflecting greater muscle torque and power—are better equipped to perform repeated sprints and achieve elevated maximum speeds, critical for their tactical role. These findings are supported by previous research demonstrating that strength qualities, particularly eccentric and concentric muscle capabilities, underpin effective sprinting and high-velocity actions in football players [36,47].

As previously mentioned, studies investigating potential correlations between strength variables and running performance indicators during football matches are extremely limited, with most of them focusing on power indicators (e.g., jump performance) rather than maximal strength indicators (e.g., isokinetic torque, one-repetition maximum). In the present study, the results were inconclusive, as the correlations varied depending on playing position, limb (dominant vs. non-dominant), and the angular velocity used in isokinetic testing. Several factors may have contributed to the inconsistent pattern of correlations. For example, isokinetic strength assessments are generally conducted using isolated, single-joint movements—such as knee extension or flexion—at predetermined, fixed angular velocities. While such protocols provide precise and reliable measurements of maximal torque under standardized conditions, they do not fully capture the functional demands of match play. In competitive football, most high-intensity actions, including sprinting, jumping, tackling, and rapid changes in direction, involve complex, multi-joint movements that require the coordinated activation of several muscle groups. These movements are typically executed at much higher velocities and under variable, unpredictable conditions. Consequently, the biomechanical and neuromuscular demands encountered on the pitch differ markedly from those assessed in the controlled environment of isokinetic testing. Additionally, the study evaluated isokinetic torque, which may not represent the critical performance factor for high-intensity match activities. Relative strength might also be a more appropriate metric, as increased muscle mass can potentially hinder performance.

Finally, this is the first study to examine these correlations while also categorizing footballers into four distinct playing positions. This positional classification is particularly important, as the tactical role and physical demands associated with each position can vary considerably. Positional differences in movement patterns inevitably influence the physical performance variables recorded, which in turn affects the strength and nature of the correlations observed. By accounting for these tactical and positional variations, the present study provides a more nuanced understanding of the relationship between physical performance metrics and the demands of competitive football.

This study had several limitations. Firstly, the sample size was small, including players from only one team, which also limited the number of players per position. Additionally, data were collected during a single season, and the team employed only one tactical formation (1-4-3-3) throughout the study period. Isokinetic torque measurements were taken only at the beginning of the season and used in analyses alongside match data collected nearly 10 months later, without repeated assessments to account for changes over time. Furthermore, torque values were not normalized to body mass, which may affect the interpretation of strength relative to player size. The isokinetic assessment was limited to a few angular velocities, and multi-joint strength tests were not included. Future research should involve larger, more diverse samples from multiple teams, incorporate different tactical formations, normalize torque data to body mass, and include multi-joint strength assessments to better understand the relationship between strength and match running performance. All the above limitations restrict the generalizability of the study’s results.

## 5. Conclusions

This study highlights that strength-performance relationships in youth soccer are both position-dependent and specific to the velocity of isokinetic assessment, yet overall, these correlations remain inconsistent. Positive associations between isokinetic strength and high-intensity running were observed in certain positions, such as SMFs, while other positions, like CM, showed negative correlations with accelerations and decelerations. Additionally, no clear links were found between strength and acceleration or deceleration metrics, suggesting that factors like rate of force development (RFD) may play a more pivotal role in these aspects of match performance.

## Data Availability

The data presented in this study are available on request from the corresponding author (due to privacy).
